# Application of Hydrate‐Melt Electrolytes to High‐Rate and High‐Capacity Organic Lithium‐Ion Batteries

**DOI:** 10.1002/cssc.70824

**Published:** 2026-07-15

**Authors:** Yoshiyuki Gambe, Saneyuki Ohno, Itaru Honma

**Affiliations:** ^1^ Institute of Multidisciplinary Research for Advanced Materials Tohoku University Sendai Miyagi Japan

**Keywords:** croconate organic cathodes, dissolution suppression, hydrate‐melt electrolytes, organic lithium‐ion batteries, sustainable energy storage

## Abstract

Lithium croconate molecules (C_5_O_5_Li_2_), a 4 V‐class high‐voltage organic cathode material, offer a possibility to develop high‐energy‐density, rare‐metal‐free, and environmentally friendly organic secondary batteries. However, organic molecules generally have a serious problem of dissolution into electrolytes, leading to poor cycling performance. Here, we demonstrate the water‐based hydrate‐melt electrolyte for improving cycling performance of organic batteries and evaluate the relationship between battery performance and solubility behavior of croconate molecules into the electrolytes. Hydrate‐melt electrolyte Li(TFSI)_0.7_(BETI)_0.3_(H_2_O)_2_ effectively suppresses the dissolution of pristine C_5_O_5_Li_2_ and C_5_O_5_ molecules (in their two‐electron charged state), leading to improved cycling performance and coulombic efficiency. This result can be ascribed to the unique solution structure having less free‐water in hydrate‐melt electrolytes. Moreover, a C_5_O_5_Li_2_/Li_4_Ti_5_O_12_ cell with hydrate‐melt electrolyte exhibits an ultrafast redox reaction with no capacity fading at higher C‐rate up to 10 C. This insight is beneficial to construct high‐power and high‐energy‐density croconate‐based storage systems.

## Graphical abstract

Stable redox reactions of lithium croconate organic cathode materials are achieved in water‐based hydrate‐melt electrolytes. The unique solution structure of hydrate‐melt electrolytes, an extremely low‐water‐activity environment, suppresses dissolution and decomposition, enabling high‐energy‐density aqueous rechargeable batteries. These findings provide a practical and broadly relevant strategy for electrolyte‐enabled stabilization of high‐voltage organic electrodes.

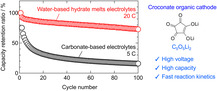

## 1 Introduction

The growing demand for batteries across a wide range of applications, from large‐scale grid storage to small‐scale portable devices, necessitates the development of cost‐effective, environmentally friendly, and resource‐abundant battery materials. In contrast, the conventional lithium‐ion batteries (LIBs) commonly rely on Ni–Mn–Co‐based oxide cathodes, which contain critical metals such as cobalt. This reliance has raised serious concerns of supply chain vulnerabilities and resource cost [1]. To address these issues, organic active materials, composed primarily of earth‐abundant light elements such as C, H, N, O, and S, have emerged as promising alternatives due to their environmental friendliness and rare‐metal‐free composition. Furthermore, many organic electrode materials can be derived from biomass [2] or synthesized under mild synthesis conditions at lower temperatures than their inorganic counterparts, resulting in a reduction of energy consumption and CO_2_ emissions. In addition, organic materials often undergo multielectron redox reactions, enabling high energy densities. To date, both small‐molecular [3, 7] and polymer‐based organic cathode materials [8, 10] have been extensively studied for next‐generation storage materials. However, only a limited number of organic cathode materials exhibit a redox potential higher than 3.0 V [11, 12].

**FIGURE 1:**
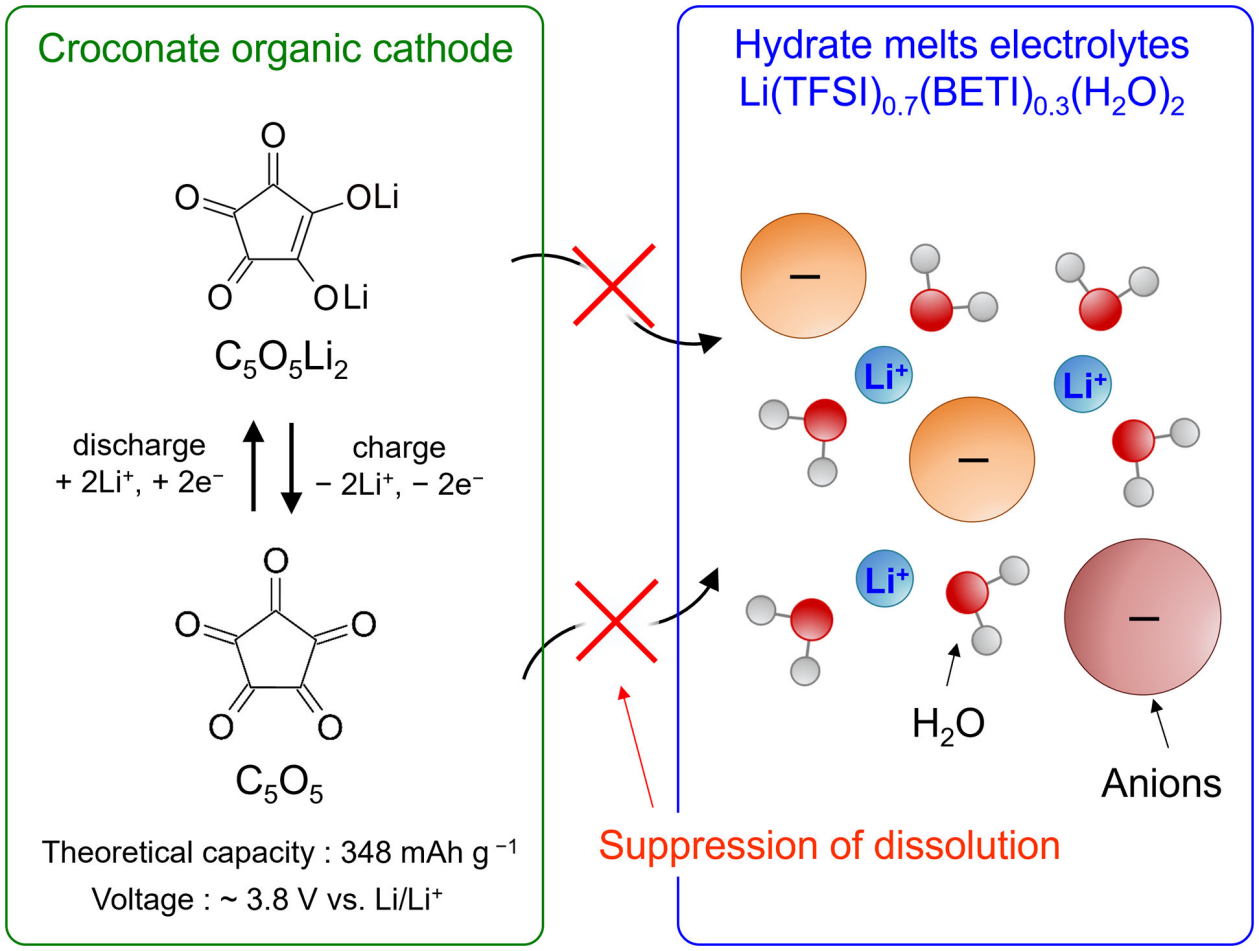
Proposed electrochemical redox reaction, theoretical capacity, and redox potential of C_5_O_5_Li_2_ molecules based on two‐electron reaction [7] and illustration of the solution structure of hydrate‐melt electrolytes.

Croconate molecules, composed of five‐membered rings with two enolate groups (–OM) and three carbonyl groups (C ═ O), have been explored as electrode materials of LIBs [13, 14], sodium‐ion batteries (SIBs) [14, 16], and potassium‐ion batteries [14]. However, previous studies primarily focused on the redox activity of carbonyl groups, which operate at voltages below 3.0 V. The redox contribution of the two enolate groups has been largely overlooked due to side reactions, such as dissolution into organic electrolytes and molecular decomposition, leading to rapid capacity fading. If the redox activity of the two lithium enolate groups (–OLi) can be fully utilized (see Figure 1), the theoretical energy density could exceed 1300 Wh kg^−1^, surpassing that of Ni–Mn–Co‐based oxide cathode. Furthermore, a high redox potential has also been demonstrated with croconate molecule‐based cathodes. In LIBs, croconic acid (C_5_O_5_H_2_) dissolved in 1 M LiPF_6_ γ‐butyrolactone exhibits redox activity at around 3.9 V [7]. In SIBs, sodium croconate (C_5_O_5_Na_2_) has been applied as a 3.5 V‐class high voltage cathode, achieving successful cycling through interfacial engineering with COOH‐functionalized multiwalled carbon nanotubes (MWCNT) via hydrogen bonding [6].

Despite these promising characteristics, a critical limitation, rapid capacity degradation over cycling, remains. This degradation is primarily attributed to the dissolution of C_5_O_5_ molecules (in their two‐electron charged state) into conventional carbonate‐based electrolytes. Moreover, C_5_O_5_ molecules can decompose into gases such as CO_2_, leading to cycling degradation. Although recent efforts, such as less‐free‐solvent electrolyte formulations using acetonitrile‐based highly concentrated electrolyte systems, have partially mitigated cycle degradation, dissolution‐induced fading remains significant [17]. Moreover, an increase in the slat concentration generally decreases ionic conductivity due to higher viscosity, resulting in poor C‐rate performance. Thus, the development of alternative electrolyte systems that can effectively suppress the solubility of C_5_O_5_ molecules and also achieve a high‐rate performance is essential to realizing the practical viability of croconate‐based organic‐ion batteries.

Recent studies suggest that the dissolution behavior of organic active materials is strongly influenced by solution structures and their interactions with electrolyte solvents [17, 18, 20], particularly through hydrogen bonding and dipolar solvation. A potential strategy to suppress such interactions is to utilize highly concentrated electrolytes in which all solvent molecules are already strongly coordinated and thus unavailable for further molecular association. This consideration brings attention to highly concentrated aqueous systems that feature reduced free (uncoordinated) water [21, 23]. Among them, a representative example is the room‐temperature hydrate melt composed of a eutectic mixture of lithium bis(trifluoromethanesulfonyl)imide (LiTFSI) and lithium bis(pentafluoroethanesulfonyl)imide (LiBETI) in a 0.7:0.3 molar ratio, with two water molecules per Li salt: Li(TFSI)_0.7_(BETI)_0.3_(H_2_O)_2_. In this system, all water molecules are tightly coordinated to Li^+^, eliminating free water and thereby lowering water activity. This unique solution structure significantly extends the electrochemical stability window beyond 3 V, enabling the operation of high‐voltage aqueous LIBs such as LiNi_0.5_Mn_1.5_O_4_/Li_4_Ti_5_O_12_ (LTO) (3.1 V) and LiCoO_2_/LTO (2.4 V). More importantly, the battery exhibited high‐rate performance at a C‐rate of 10 C [23]. In addition, highly concentrated aqueous electrolytes have also been applied to Zn‐ion battery systems in recent years [24, 25].

Inspired by the aforementioned experimental evidence, we hypothesize that the coordination‐saturated and extremely low‐water‐activity environment of hydrate‐melt electrolytes could serve as an effective medium to suppress the dissolution of organic cathode materials by fundamentally minimizing solute–solvent interactions, thereby achieving ultrahigh‐rate performance. In addition, only very limited studies have reported the application of highly concentrated aqueous electrolytes to small organic cathode materials [26], and the interfacial stability in such system remains largely unexplored. Hence, in this work, we investigate the water‐based hydrate‐melt electrolyte for improving cycling properties of organic LIBs with croconate cathode and study the relationship between battery performance and dissolution behavior in carbonate‐based organic electrolytes and hydrate‐melt electrolytes. In general, organic molecules dissolve into electrolytes through interactions with solvents such as hydrogen bonding and polarity. Hydrate‐melt electrolytes possess a unique solution structure with reduced free‐water activity, thereby weakening the interaction between C_5_O_5_ molecules (in their two‐electron charged state) and the water solvent, which is expected to suppress the dissolution and decomposition of croconate cathode molecules and improve the cycling performance. The solubility behavior of pristine C_5_O_5_Li_2_ molecules and C_5_O_5_ molecules is examined by UV–vis and ^13^C‐NMR techniques, and, moreover, the rapid reaction kinetics between the croconate cathode and hydrate‐melt electrolyte are demonstrated in this work.

## 2 Results and Discussion

The C_5_O_5_Li_2_ powder was prepared by a neutralization reaction of C_5_O_5_H_2_ and LiOH in aqueous solution by the reprecipitation method. The crystal structures and microstructure of synthesized C_5_O_5_Li_2_ molecules were evaluated by X‐ray diffraction (XRD) (see Figure S1) and scanning electron microscopy (SEM) image (see Figure S2). The XRD pattern of the C_5_O_5_Li_2_ molecules is in good agreement in previous reports [14], and no peaks of raw materials such as C_5_O_5_H_2_ and LiOH were observed. The particle size of the synthesized C_5_O_5_Li_2_ molecules is smaller than 1 µm.

**FIGURE 2:**
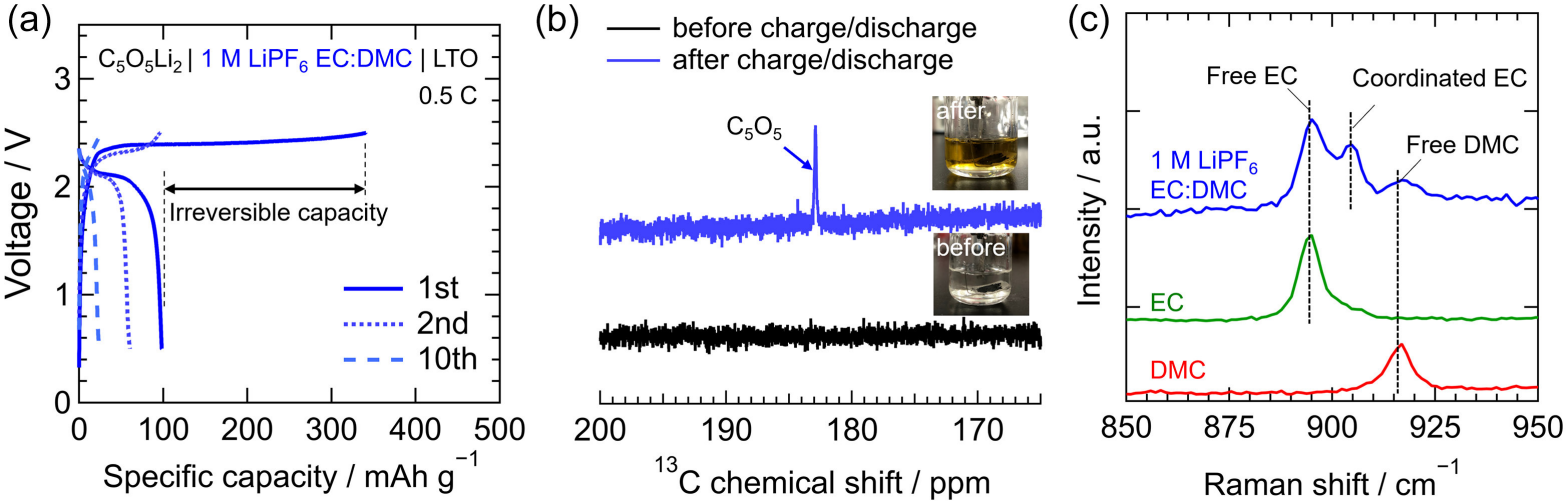
(a) First, second, and 10th charge–discharge profiles of C_5_O_5_Li_2_/LTO batteries with 1 M LiPF_6_ EC:DMC (1:1, v/v) at a C‐rate of 0.5 C. The first irreversible capacity was indicated. (b) ^13^C‐NMR spectra of 1 M LiPF_6_ EC:DMC (1:1, v/v) before and after first charge–discharge measurements. Photographs of each electrolyte before and after first charge–discharge measurements are inserted. (c) Raman spectra of 1 M LiPF_6_ EC:DMC (1:1, v/v), EC solvent, and DMC solvent.

First, to evaluate the redox reaction of the C_5_O_5_Li_2_ cathode, the battery performance of a C_5_O_5_Li_2_/LTO cell with carbonate‐based organic electrolyte was assessed at a C‐rate of 0.5 C (see Figure 2a). The LTO anode was selected to properly compare the cathode performance with organic electrolytes and water‐based hydrate melts. The capacity of 340 mAh g^−1^, which is comparable to the theoretical capacity of C_5_O_5_Li_2_ (348 mAh g^−1^), was delivered upon the first charging, indicating that almost all C_5_O_5_Li_2_ molecules were converted to C_5_O_5_ molecules; the two‐electron oxidation reaction of lithium enolate (–OLi) in C_5_O_5_Li_2_ molecules proceeds. However, only 29% (98 mAh g^−1^) of the first charging capacity was obtained in the first discharge capacity. Rapid decay resulted in a 10th discharge capacity of 24 mAh g^−1^, highlighting poor reversibility with carbonate‐based organic electrolytes. The ^13^C‐NMR spectra of the electrolytes before and after the first cycle shown in Figure 2b revealed that a peak at approximately 183 ppm, which is attributable to the C_5_O_5_ molecule [27], appeared after the first cycle. The electrolyte color has also changed to yellow after the first cycle, confirming the dissolution of the organic molecules. From the result of Raman spectra of carbonate‐based organic electrolytes (see Figure 2c), free (noncoordinating to Li^+^) ethylene carbonate (EC) and dimethyl carbonate (DMC) solvents remain in the 1 M LiPF_6_ EC:DMC electrolytes [28], and C_5_O_5_ molecules interact with free solvents, resulting in the dissolution of organic active materials. These results highlight that rapid capacity decay originates from the loss of active materials in C_5_O_5_ form, dissolving into carbonate‐based organic electrolytes. To improve the battery performance with C_5_O_5_Li_2_ organic cathode, we evaluated water‐based hydrate‐melt electrolytes in the following section.

**FIGURE 3:**
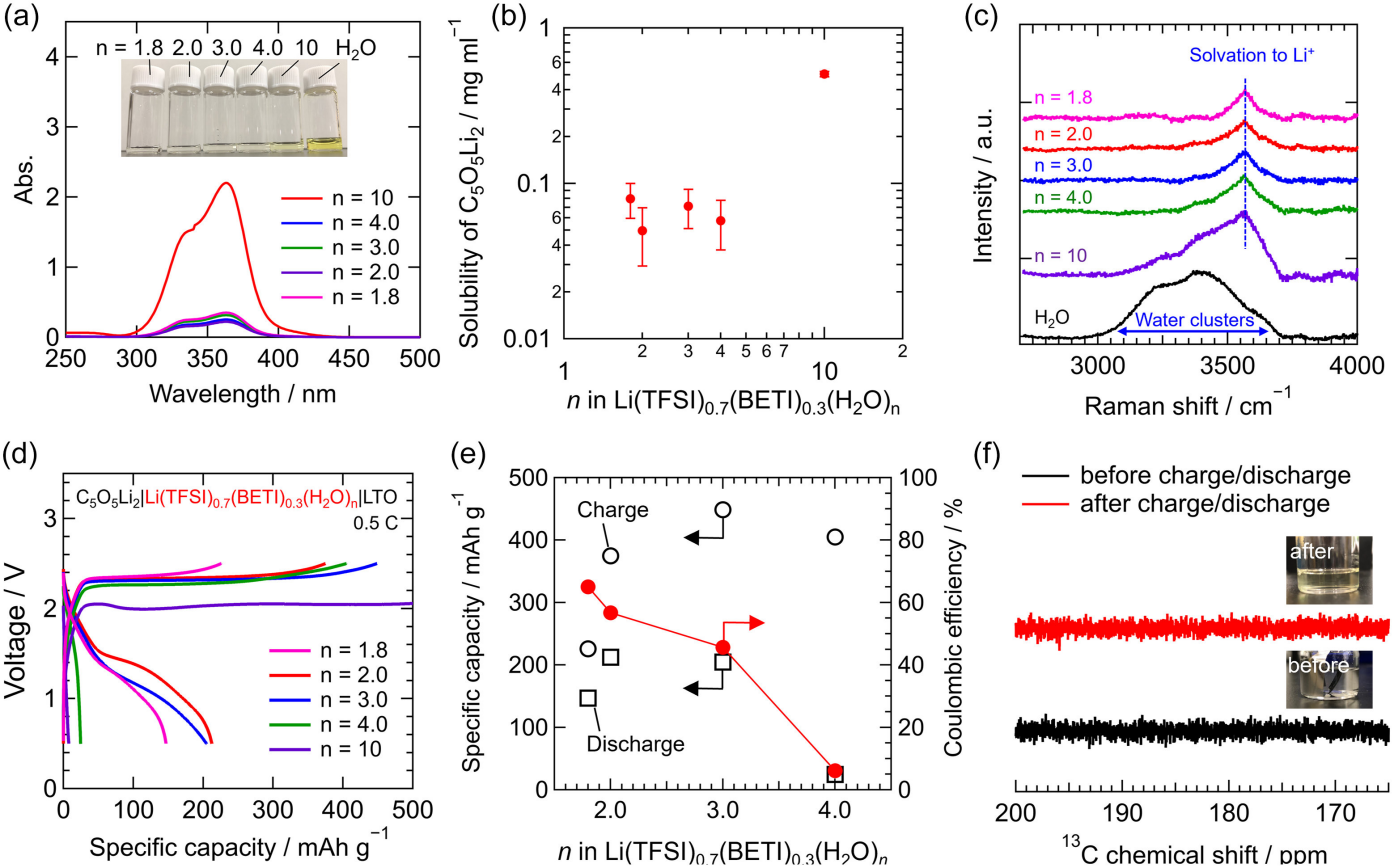
(a) UV–vis spectra of the electrolytes after filtration, which was diluted 40 times with pure water solvent. Photographs of the dissolution behavior of C_5_O_5_Li_2_ powder in Li(TFSI)_0.7_(BETI)_0.3_(H_2_O)_*n*_ (*n* = 1.8, 2.0, 3.0, 4.0, and 10) and H_2_O solvent after filtration were inserted. (b) Solubilities of the C_5_O_5_Li_2_ powder into Li(TFSI)_0.7_(BETI)_0.3_(H_2_O)_*n*_ (*n* = 1.8, 2.0, 3.0, 4.0, and 10) calculated from the standard curve at 363 nm as shown in Figure S4. The error bars correspond to 99% confidence intervals. (c) Raman spectra of Li(TFSI)_0.7_(BETI)_0.3_(H_2_O)_*n*_ (*n* = 1.8, 2.0, 3.0, 4.0, and 10) and H_2_O solvent. (d) First charge–discharge profiles of C_5_O_5_Li_2_/LTO batteries with Li(TFSI)_0.7_(BETI)_0.3_(H_2_O)_*n*_ (*n* = 1.8, 2.0, 3.0, 4.0, and 10) at a C‐rate of 0.5 C. (e) First charge and discharge capacities, and coulombic efficiencies were plotted as a function of Li‐salt concentration. (f) ^13^C‐NMR spectra of electrolytes of Li(TFSI)_0.7_(BETI)_0.3_(H_2_O)_2_ before and after first charge–discharge measurements. Photographs of each electrolyte before and after first charge–discharge measurements are inserted.

To evaluate the effect of the Li‐salt concentration, the solubility behavior of pristine C_5_O_5_Li_2_ molecules into water‐based electrolytes was evaluated using UV–vis techniques (see Figure 3a,b). Pristine C_5_O_5_Li_2_ powder before charge–discharge measurements easily dissolves into pure water due to its high polarity. On the other hand, it was confirmed that the fraction of C_5_O_5_Li_2_ powder that dissolves into the water‐based electrolytes (Li(TFSI)_0.7_(BETI)_0.3_(H_2_O)_*n*_, *n* = 1.8–10) is drastically reduced as the Li‐salt concentration increases (see Figure S3). After filtration of that solution, UV–vis measurements were conducted, and absorbance spectra at approximately 363 nm, derived from C_5_O_5_Li_2_ molecules, were observed (see Figure 3a). Although the absorbance was higher in a *n* = 10 of Li(TFSI)_0.7_(BETI)_0.3_(H_2_O)_*n*_ solution, for the other samples with higher Li‐salt concentrations (*n* = 1.8–4.0), the absorbance decreased (see Figure 3a). The solubilities of the C_5_O_5_Li_2_ powder were calculated from the calibration curve at 363 nm, which showed a linear relationship (see Figure S4). The solubility of the water‐based electrolyte in *n* = 10 was 0.51 mg ml^−1^, whereas the other samples had lower solubility than 0.1 mg ml^−1^ (see Figure 3b).

To further discuss the solubility behavior, the solution structure of water‐based electrolytes was evaluated by Raman spectra (see Figure 3c). A broadband from 2950 to 3700 cm^−1^, which corresponds to the various hydrogen bonding environments in water clusters [23], was observed in pure H_2_O solvent. The intensity of the hydrogen‐bond‐derived Raman band was decreased by increasing Li‐salt concentrations. Below *n* = 4 (*n* = 1.8 – 4.0), the Raman bands associated with hydrogen bonding in bulk‐water clusters disappeared, whereas only a peak at approximately 3565 cm^−1^, corresponding to Li^+^‐solvated water molecules, was observed, implying that the amount of water clusters (free water) was extremely decreased. The compositional threshold (*n* = 4) coincides with the solubility trend, suggesting a correlation with a Li^+^ coordination number of approximately four [29]. From the above results, an increase in Li‐salt concentration could reduce the activity of water molecules as reported in a previous study [23, 30], indicating that the solubility of C_5_O_5_Li_2_ powder into the hydrate‐melt electrolytes drastically decreased from water‐cluster‐rich electrolyte (*n* = 10) to highly coordinated hydrate‐melt structure (*n* < 4). Once this coordinated solvation structure is established, further decrease of water activity results in only a limited additional decrease of solubility, leading to the observed plateau‐like solubility behavior below *n* = 4 in Figure 3b. In this study, the electrolyte of 40 µL and the C_5_O_5_Li_2_ powder active material of 0.7 mg (17.5 mg mL^−1^) were used for the evaluation of battery performance, and it was found that the effect of dissolution of pristine C_5_O_5_Li_2_ molecules into the Li(TFSI)_0.7_(BETI)_0.3_(H_2_O)_*n*_ (*n* = 2) is eliminated.

On the basis of solubility behavior, the battery performance of a C_5_O_5_Li_2_/LTO cell with Li(TFSI)_0.7_(BETI)_0.3_(H_2_O)_*n*_ (*n* = 1.8, 2.0, 3.0, 4.0, and 10) was evaluated at a C‐rate of 0.5 C (see Figure 3d). To focus on the intrinsic electrochemical behavior and electrolyte compatibility of the C_5_O_5_Li_2_ organic active material, cathodes composition with a relatively high conductive‐carbon content was employed in this study. The electrode used in this study had an active‐materials loading of 1.8 mg cm^−2^ and an electrode thickness of 150 µm. For a *n* = 10 in Li(TFSI)_0.7_(BETI)_0.3_(H_2_O)_*n*_, a side reaction was observed at approximately 2.0 V, indicating the remaining free‐water in the electrolyte (as discussed in Figure 3c) was reduced and decomposed at the LTO anode side. Below *n* = 4, charging profiles at approximately 2.4 V (3.9 V vs. Li/Li^+^) were observed; the oxidation of lithium enolate (–OLi) in C_5_O_5_Li_2_ molecules proceeded with a similarity to the result for carbonate‐based organic electrolytes in Figure 2a. Moreover, at *n* = 2.0, the capacity of 212 mAh g^−1^ was delivered upon the first discharging, which is higher than that of organic electrolytes. To clearly discuss the correlation between the battery performances and Li‐salt concentrations, the first charge and discharge capacities, and first coulombic efficiencies (CE) were summarized as a function of Li‐salt concentration in Figure 3e. The CE was improved to 65% as the Li‐salt concentration increased to *n* = 1.8, although the specific capacity decreased at the composition of *n* = 1.8 due to an increase in overpotential with increased viscosity of the electrolyte. By contrast, the first charge and discharge capacities of the cells with Li(TFSI)_0.7_(BETI)_0.3_(H_2_O)_*n*_ (*n* = 2) were 374 and 212 mAh g^−1^, respectively, resulting in the first CE improved to 57%, which is higher than that of the cell with carbonate‐based organic electrolytes. It was revealed that the cell with Li(TFSI)_0.7_(BETI)_0.3_(H_2_O)_*n*_ (*n* = 2) exhibited better battery performance. Yet a quick capacity fade was still observed at a C‐rate of 0.5 C (see Figure S5).

From the result of ^13^C‐NMR measurement (see Figure 3f), no ^13^C‐NMR peak was observed in the range from 165 to 200 ppm, indicating that water‐based hydrate‐melt electrolytes suppressed the dissolution of C_5_O_5_ molecules (two‐electron charged state) and also pristine C_5_O_5_Li_2_ molecules (see Figure S6). Although the color of the water‐based hydrate‐melt electrolytes slightly changed to a light yellow after charge–discharge measurements, we consider that the color would change due to the hydrolysis of a small amount of C_5_O_5_ molecules. Based on the above results, it was confirmed that the hydrate‐melt electrolyte effectively suppresses the dissolution of pristine C_5_O_5_Li_2_ and C_5_O_5_ molecules, owing to its unique solution structure with extremely low free water, thereby improving CE.

While the effect of the dissolution of molecules was almost negligible based on the above results of dissolution behavior of croconate molecules (see Figure 3b,f), the capacity decay is still significant at lower C‐rates of 0.5 C (see Figure S5). Previous studies on C_*n*_O_*n*_ molecules (*n* = 4, 6) have reported hydration‐related structural change in aqueous environments, where carbonyl groups (C ═ O) are changed to hydroxylated structure [31]. Because the carbonyl groups serve as electrochemically redox‐active sites, above structural transformations can result in the loss electrochemical activity. Thus, although similar reaction for C_5_O_5_ molecules have not yet been directly clarified, other capacity degradation mechanisms, such as hydration‐related hydroxylation and/or hydrolysis of C_5_O_5_ in the presence of extremely low levels of residual free water and nanoclusters of water in hydrate‐melt electrolytes [32], may be considerable. Although possible parasitic reactions at the LTO/aqueous electrolyte interface cannot be completely excluded, the relatively stable electrochemical behavior of LTO anode in hydrate‐melt electrolyte suggests that severe interfacial degradation is suppressed under the present conditions. In the following, the impact of further reduction in the content of free water was explored by using another eutectic system with asymmetric imide anion to further improve the cycling performance. We focused on the lithium‐salt monohydrate melt electrolyte composed of a eutectic mixture of lithium (trifluoromethanesulfonyl)(pentafluoroethanesulfonyl) imide (LiPTFSI) and LiTFSI in a 0.6:0.4 molar ratio, with one water molecules per Li‐salt: Li(PTFSI)_0.6_(TFSI)_0.4_(H_2_O) [30]. Although *n* = 1.0 is ideal, *n* = 1.2 in Li(PTFSI)_0.6_(TFSI)_0.4_(H_2_O)_*n*_ (see Figure S7) with extremely reduced free‐water (see Raman spectrum in Figure S8) was employed for battery operation in this study as it ensures complete dissolution of Li‐salts. The first charge and discharge capacities of the cells with Li(PTFSI)_0.6_(TFSI)_0.4_(H_2_O)_1.2_ were 321 and 171 mAh g^−1^, respectively, resulting in the first CE of 53% (see Figure S9a), which is comparable to that with Li(TFSI)_0.7_(BETI)_0.3_(H_2_O)_2_ (see Figure 3d). Furthermore, the capacity of 88 mAh g^−1^ was delivered upon the 50th discharging, resulting in the capacity retention ratio of 51%. The performance of the cells with Li(PTFSI)_0.6_(TFSI)_0.4_(H_2_O)_1.2_ was superior to those of carbonate‐based organic electrolytes and Li(TFSI)_0.7_(BETI)_0.3_(H_2_O)_2_ electrolytes (see Figure S9b); the hydration‐related hydroxylation and/or hydrolysis of C_5_O_5_ could be suppressed using Li(PTFSI)_0.6_(TFSI)_0.4_(H_2_O)_1.2_ with lower water activity. In contrast, capacity fading at higher C‐rates was observed as shown in Figure S10 because the viscosity is drastically increased to 1426 cP at 25°C (see Figure S11), resulting in low ionic conductivity [30]. From the above results, it was revealed that insoluble properties in the electrolytes and chemical compatibility between electrolytes and C_5_O_5_ molecules are both essential to construct more stable croconate‐based organic LIBs. In addition to low water activity of highly concentrated aqueous electrolytes, a high ionic transference property is also crucial for high‐rate battery performance.

**FIGURE 4:**
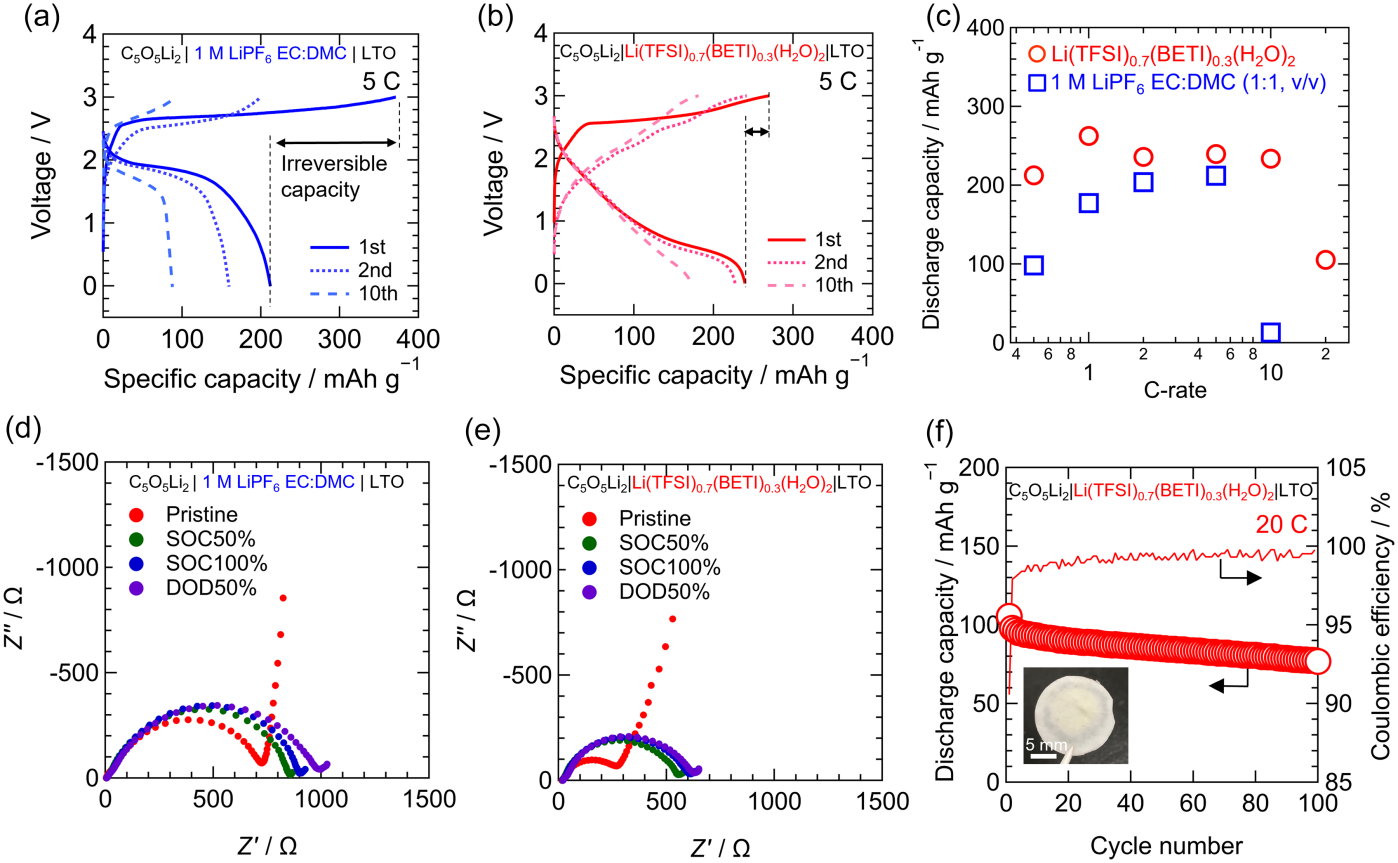
(a) First, second, and 10th charge–discharge profiles of C_5_O_5_Li_2_/LTO batteries with (a) 1 M LiPF_6_ EC:DMC (1:1, v/v) and (b) Li(TFSI)_0.7_(BETI)_0.3_(H_2_O)_2_ at a C‐rate of 5 C and 25°C. First irreversible capacity was indicated. (c) First discharge capacities of the croconate‐based organic LIBs with Li(TFSI)_0.7_(BETI)_0.3_(H_2_O)_2_ and 1 M LiPF_6_ EC:DMC (1:1, v/v) at C‐rates from 0.5 C to 20 C and 25°C. Nyquist plots of the croconate‐based organic LIBs with (d) 1 M LiPF_6_ EC:DMC (1:1, v/v) and (e) Li(TFSI)_0.7_(BETI)_0.3_(H_2_O)_2_ before cycling (pristine), and state of charge (SOC) 50%, SOC100%, and depth of discharge (DOD) 50% during first charge–discharge measurement. (f) Discharge capacity and CE of the cells with Li(TFSI)_0.7_(BETI)_0.3_(H_2_O)_2_ at a C‐rate of 20 C and a photograph of the separator after 100 cycles was inserted.

Finally, to evaluate the fast reaction kinetics of C_5_O_5_Li_2_‐based organic LIBs with hydrate‐melt electrolytes (Li(TFSI)_0.7_(BETI)_0.3_(H_2_O)_2_), having higher ionic conductivity, the C‐rate properties of the cells were evaluated. The first irreversible capacity of the cell with hydrate‐melt electrolytes drastically decreased compared to that with carbonate‐based organic electrolytes at a high C‐rate of 5 C (see Figure 4a,b). In contrast, the relatively large polarization was observed after the second cycle, as shown in Figure 4b. To clarify the origin of the polarization after the second cycle, ex situ XRD measurements were conducted on the C_5_O_5_Li_2_ electrode before and after cycling (see Figure S12). The characteristic diffraction pattern of pristine C_5_O_5_Li_2_ almost disappeared after the second cycle, while several new diffraction peaks were observed, indicating structural changes during cycling in the hydrate‐melt aqueous electrolyte. To further investigate the structural changes, hydrated C_5_O_5_Li_2_, the intermediate product formed before dehydration at 150°C during the synthesis of C_5_O_5_Li_2_, was prepared, and XRD and thermogravimetric (TG) measurements were conducted (see Figure S13). TG analysis revealed that the obtained hydrate phase corresponded to C_5_O_5_Li_2_·2H_2_O. Importantly, the diffraction peaks of the hydrated phase were in good agreement with those observed for the second cycled electrode, as shown in Figure S12b. These results suggest that structural change associated with hydrate formation occurs during charge–discharge cycling in the hydrate‐melt aqueous electrolyte. We therefore consider that the formation of the hydrated organic active‐material phase (C_5_O_5_Li_2_·2H_2_O) alters the thermodynamic redox potential of the active material, leading to changes in the voltage profile and increased overpotential after the second cycle. While there is no experimental evidence, there is also a chance of slow Li‐ion diffusion kinetics in the hydrated phase, contributing to polarization at high rates. Meanwhile, the impact of hydroxylation and/or hydrolysis of C_5_O_5_ molecules could be suppressed in this time scale of 5 C, delivering a high first CE of 89% (see Figure 4b). Moreover, the capacity of 172 mAh g^−1^ was delivered upon the 10th discharging (see Figure 4b), resulting in the capacity retention ratio of 72%.

To further evaluate the practical electrochemical performance, the average charge/discharge voltages, voltage efficiency (VE), CE, and energy efficiency (EE) were compared between the hydrate‐melt electrolyte and conventional organic electrolyte systems during the first cycle. The hydrate‐melt electrolyte system exhibited average first charge and discharge voltages of 2.64 and 1.02 V, respectively, whereas the organic electrolytes system showed average first charge and discharge voltages of 2.72 and 1.61 V, respectively. Consequently, the VE of hydrate‐melt electrolyte system (38%) was lower than that of the organic electrolyte system (59%). Ex situ XRD analysis suggested that structural changes associated with hydrate formation occur during cycling, which might alter the redox potential and induce low Li‐ion diffusion kinetics, contributing to increased polarization and reduced VE. In contrast, the hydrate‐melt electrolyte exhibited a significantly higher CE (89%) than the organic electrolyte system (57%), indicating more effective mitigation of dissolution and parasitic side reactions, such as hydroxylation and/or hydrolysis of C_5_O_5_ molecules. As a result, the EE values of the hydrate‐melt and the organic electrolyte systems were nearly identical at approximately 34% under the present conditions. These results suggest that the hydrate‐melt electrolyte improves the reversibility of the electrochemical reaction, although further optimization of the polarization behavior will be necessary to improve the practical EE.

The first discharge capacities of the cell with 1 M LiPF_6_ EC:DMC electrolytes were 177, 203, 212, and 12 mAh g^−1^, at C‐rates of 1 C, 2 C, 5 C, and 10 C, respectively (see Figure 4c). The cycle degradation, arising from the dissolution of C_5_O_5_ molecules, was observed as shown in Figure S14. At a higher C‐rate of 10 C, the discharge capacity suddenly decreases due to high overpotential (see Figure S14c). In contrast, the first discharge capacity at a C‐rate of 10 C was 233 mAh g^−1^, almost no capacity fading from 0.5 C up to higher C‐rate of 10 C (see Figure 4c and Figure S15). The C_5_O_5_Li_2_‐based organic LIBs with hydrate‐melt electrolytes showed an excellent high‐rate performance. Impedance measurements shown in Figure 4d,e indicate that the hydrate‐melt electrolyte system exhibits relatively low charge‐transfer resistance, suggesting favorable interfacial charge‐transfer behavior in the aqueous highly concentrated environment. In contrast, as discussed above, the thermodynamic redox‐potential changes and lower Li‐ion diffusion kinetics may become dominant factors of the electrochemical overpotential during cycling. Nevertheless, the C_5_O_5_Li_2_ electrode still exhibited relatively stable high‐rate performance up to 10 C, suggesting the hydrated organic active‐material phase remains intrinsically fast redox reaction even after the structural change. Considering the facts that high C‐rate performances in the C_6_O_6_K_2_ and C_5_O_5_Na_2_ cathodes in K‐ion and Na‐ion batteries [14, 17], these results highlight the intrinsic nature of the two lithium enolate groups (–OLi) in C_5_O_5_Li_2_ molecules may pave the way for ultrafast redox reaction kinetics. Therefore, further molecular and crystal‐structure design to eliminate hydrate formation could be an effective strategy for improving VE and high‐rate performance.

As for the capacity retention, the discharge capacity retention ratio was exhibited to 73% after 100 cycles at a higher C‐rate of 20 C, corresponding to a current density of 12.8 mA cm^−2^ (see Figure 4f), and the cycling performance of the C_5_O_5_Li_2_‐based organic LIBs with hydrate‐melt electrolytes was drastically improved. The first discharge capacity and areal capacity were 105 mAh g^−1^ and 0.19 mAh cm^−2^, respectively. Almost no color change of the separator after 100 cycles was observed (see inset in Figure 4f); the dissolution of C_5_O_5_ molecules was suppressed, which is consistent with the result of ^13^C‐NMR (see Figure 3f). The performance of the croconate‐based organic batteries in this study was superior to previous results (summarized in Table S1). Based on the highly concentrated electrolytes as an approach toward stable organic batteries, the suppression of free water activity and the formation of a Li^+^‐centered coordination environment effectively reduced dissolution and degradation of croconate organic molecules, leading to a stable electrochemical performance at even higher C‐rate of 20 C. These findings highlight highly concentrated aqueous electrolytes as a promising medium for high‐power, precious‐metal‐free organic LIBs and provide important insight for the rational design of electrolyte systems for organic electrode materials.

## 3 Conclusion

In summary, we demonstrated hydrate‐melt electrolytes for croconate‐based organic LIBs and evaluated the relationship between the battery performance and the dissolution behavior of organic molecules. The hydrate‐melt electrolytes Li(TFSI)_0.7_(BETI)_0.3_(H_2_O)_2_ effectively suppressed the dissolution of pristine C_5_O_5_Li_2_ and C_5_O_5_ (in their two‐electron charged state) molecules due to the unique solution structure with significantly reduced free‐water activity, leading to better cycling performance and CE. We also evaluated the ultrahigh C‐rate performance of the cells with hydrate‐melt electrolytes. The cell exhibited an ultrafast redox reaction with no capacity fading from 0.5 C up to higher C‐rate of 10 C, and a high cycling retention ratio of 73% after 100 cycles at an extremely high C‐rate of 20 C.

## Funding

This work was supported by the JSPS KAKENHI (21H04696 and 25H00207), Toyota Physical and Chemical Research Institute through the Rising Fellow Program.

## Conflicts of Interest

The authors declare no conflicts of interest.

## Supporting information

Supplementary Material

## Data Availability

The data that support the findings of this study are available from the corresponding author upon reasonable request.
