# Supplementary material for: Application of Hydrate‐Melt Electrolytes to High‐Rate and High‐Capacity Organic Lithium‐Ion Batteries
Source: ChemSusChem. 2026 Jul 15;19(14):e70824. doi: 10.1002/cssc.70824 (PMC13372234; doi:10.1002/cssc.70824)
Supplement: Supplementary file 1 — Supplementary Material [file CSSC-19-e70824-s001.pdf]

Supporting Information

Application of hydrate-melt electrolytes to high-rate and high-capacity organic lithium-ion batteries

Yoshiyuki Gambe,[a] Saneyuki Ohno,[a] Itaru Honma[a]*

[a]Institute of Multidisciplinary Research for Advanced Materials, Tohoku University,

2-1-1 Katahira, Aoba-ku, Sendai, Miyagi 980-8577, Japan

*Corresponding author

E-mail: itaru.homma.e8@tohoku.ac.jp

Experimental Section

Materials

$C_5O_5H_2$ (>98%, Tokyo Chemical Industry Co., Ltd.) and LiOH (>98%, anhydrous, Tokyo Chemical Industry Co., Ltd.) were used to synthesize $C_5O_5Li_2$ powder by neutralization method. 100 mM LiOH solution (10 ml) was added slowly into 50 mM $C_5O_5H_2$ solution (10 ml) and mixed for 1 h. The resultant solution was rapidly injected into 400 mL acetone with a 22 G needle, the C_5O_5Li crystal was collected through filtration and washed by acetone five times. To remove the crystal water, the $C_5O_5Li_2$ powder was dried at 150°C under vacuum for 12 h. COOH-MWCNT (COOH functionalized: >8%, average diameter: 9.5 nm, length: 1.5 μm, Sigma-Aldrich Co.) and polytetrafluoroethylene (PTFE, Teflon-6J, DuPont-Mitsui Fluorochemicals Co., Ltd.) were used as conductive carbon and binder, respectively. $Li_4Ti_5O_{12}$ (LTO, LT-105, Ishihara Sangyo Kaisha Ltd.), acetylene black (AB, FX-35, Denki Kagaku Kogyo KK), and polyvinylidene difluoride (PVDF, KF-1100, KUREHA) were used as anode active material, conductive carbon, and binder for anode sheet, respectively. Lithium bis(trifluoromethanesulfonyl)imide (LiTFSI, 99.9%, Kishida Chemicals Co., Ltd.) and lithium bis(pentafluoroethanesulfonyl)imide (LiBETI, >98.0%, Tokyo Chemical Industry Co., Ltd.) were used to prepare water-based electrolytes. Lithium (trifluoromethanesulfonyl)(pentafluoroethanesulfonyl) imide (LiPTFSI) was provided by Mitsubishi Materials Electronic Chemicals. The electrolytes solution was prepared by dissolving LiTFSI and LiBETI salts into ultrapure water at molar ratios of LiTFSI:LiBETI:H_2O = 0.7:0.3:n (n = 1.8, 2.0, 3.0, 4.0, and 10). 1 M $LiPF_6$ ethylene carbonate: dimethyl carbonate (EC:DMC = 1:1, v/v, Kishida Chemicals Co., Ltd.) was used as an organic electrolyte. The other type of highly concentrated aqueous electrolyte was prepared by dissolving LiPTFSI and LiTFSI salts into pure water at molar ratios of LiPTFE:LiTFSI:H_2O = 0.6:0.4:n (n = 1.2, and 2.0).

Characterizations

The crystal structure and microstructure of the synthesized $C_5O_5Li_2$ organic material were evaluated by X-ray diffractometer (XRD, SmartLab 3G, Rigaku, Cu Kα irradiation source) and field-emission scanning electron microscopy (FE-SEM, JSM-7800F, JEOL Ltd.), respectively. The solubility of $C_5O_5Li_2$ molecules into water-based electrolytes was evaluated by ultraviolet-visible spectroscopy (UV-Vis, UVN-6700 Spectrophotometer, JASCO). The powder of $C_5O_5Li_2$ (3 mg) was mixed with $Li(TFSI)_{0.7}(BETI)_{0.3}(H_2O)_n$ electrolytes (500 μl, n = 1.8, 2.0, 3.0, 4.0, and 10) at 50°C in 1 h. The resultant dispersion was filtered by PTFE membrane (ADVANTEC, 0.45 μm) and the filtrates diluted by ultrapure water were characterized by UV-Vis spectrometer with a wavelength range of 250-500 nm. The chemical structures of organic molecules dissolved into electrolytes were characterized by ^{13}C Nuclear Magnetic Resonance spectroscopy (^{13}C-NMR, AVANCE IV, 500 MHz). Deuterated dimethyl sulfoxide (DMSO-d_6) was used as a solvent and ^{13}C chemical shift was calibrated to the residual DMSO-d_6 peak at 39.5 ppm. The solution structure of the electrolytes was evaluated by Raman spectroscopy (Renishaw InVia Qontor, excitation wavenumber = 532 nm). The viscosities of the aqueous electrolytes were determined using a Brookfield viscometer (DV2T; LV). The structural change of $C_5O_5Li_2$ cathode after charge-discharge cycling was evaluated by ex situ XRD measurement. A coin cell of $C_5O_5Li_2$/LTO full-cell after second cycling was disassembled in Ar-filled glovebox, and the $C_5O_5Li_2$ cathode sheet was prepared by completely washing with dimethyl carbonate and drying at room temperature under vacuum. The cathode sheets before and after second charge-discharge cycling were sealed using polyimide film, and ex situ XRD measurement was carried out without exposure to air.

Battery performance of $C_5O_5Li_2$-based LIBs

A cathode sheet was prepared by mixing the $C_5O_5Li_2$, COOH-MWCNT, and PTFE using an agate mortar and pestle, with a weight ratio of 23.7:71.3:5.0. The cathode sheet (diameter = 7 mm) was prepared, and the cathode weight was adjusted to approximately 3.0 mg. The COOH-MWCNT carbon material was used as conductive additive as reported in our recent research. To prepare LTO anode film, the LTO powder and AB were well-mixed, and the resultant powder was dispersed in PVDF/*N*-methylpyrrolidone solution by using a planetary centrifugal mixer (AR-100 conditioning mixier, Thinky). The resultant slurry was coated onto Al foil as a current collector and dried at 120°C under vacuum 12 h. The weight ratios of LTO, AB, and PVDF were 80, 10, and 10 wt%, respectively. The electrode loading density is approximately 4-5 mg cm^{-2}. A cell was fabricated by stacking the $C_5O_5Li_2$ cathode sheet, a glass fiber (GA55, ADVANTEC) as the separator, and the LTO anode sheet in a CR2032-type coin cell in an Ar-filled glove box. The water-based electrolytes (40 μL) or 1 M $LiPF_6$ EC:DMC organic electrolytes (40 μL) were soaked into the separator. A Ti foil (diameter = 16 mm) was inserted between the $C_5O_5Li_2$ cathode sheet and SUS-based coin-cell cap to suppress the corrosion of the SUS. An Al foil (diameter = 16 mm) was inserted between the LTO anode sheet and SUS-based spacer. The capacity of the negative electrode to the positive electrode (N/P ratio) was fixed at approximately 1.7-2.0. The specific capacities of the cells were calculated based on the mass of $C_5O_5Li_2$ in the cathode composite sheet. The charge-discharge measurements were carried out at 25°C and C-rates of 0.5–20 C in the cut-off voltage range of 0.5–2.5 V or 0.0–3.0 V.

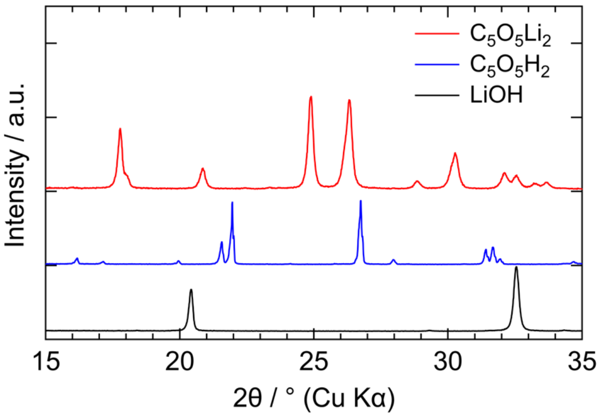

Figure S1. XRD patterns of syntheseized $C_5O_5Li_2$ powder, and $C_5O_5H_2$ powder and LiOH powder as raw materials.

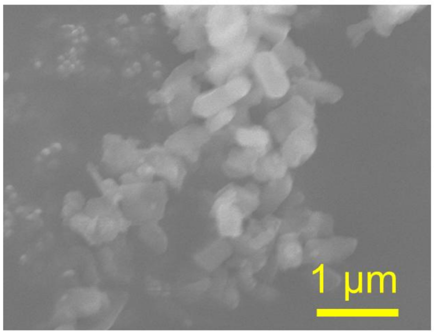

Figure S2. SEM image of $C_5O_5Li_2$ powder.

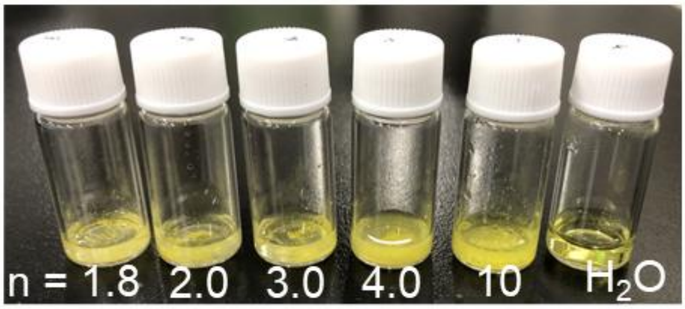

Figure S3. Photographs of the dissolution behavior of $C_5O_5Li_2$ powder (3 mg) in $Li(TFSI)_{0.7}(BETI)_{0.3}(H_2O)_n$ (n = 1.8, 2.0, 3.0, 4.0, and 10) and H_2O solvent (500 μl).

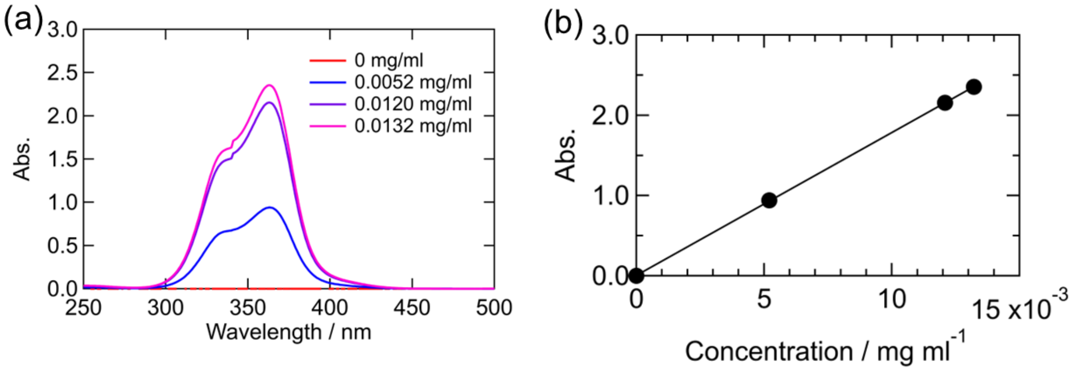

Figure S4. (a) UV-Vis spectra of reference aqueous solution. $C_5O_5Li_2$ powder was dissolved into ultra-pure water with concentrations of 0, 0.0052, 0.0120, and 0.0132 mg ml^{-1}. (b) Calibration curve of $C_5O_5Li_2$ was obtained at 362 nm absorbance of $C_5O_5Li_2$ dissolved in the pure water and provide a linear relationship between the concentration and absorbance.

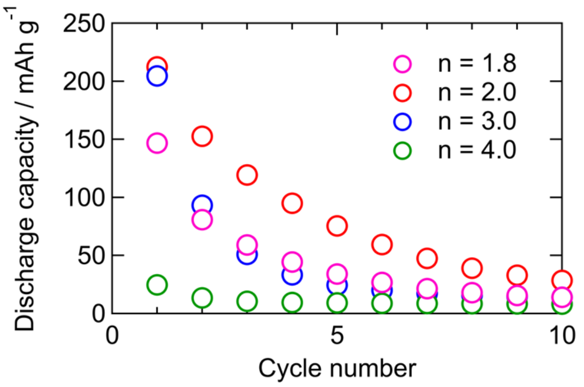

Figure S5. Cycle performance of the $C_5O_5Li_2$-based organic LIBs with $Li(TFSI)_{0.7}(BETI)_{0.3}(H_2O)_n$ (n = 1.8, 2.0, 3.0, and 4.0) at a C-rates of 0.5 C.

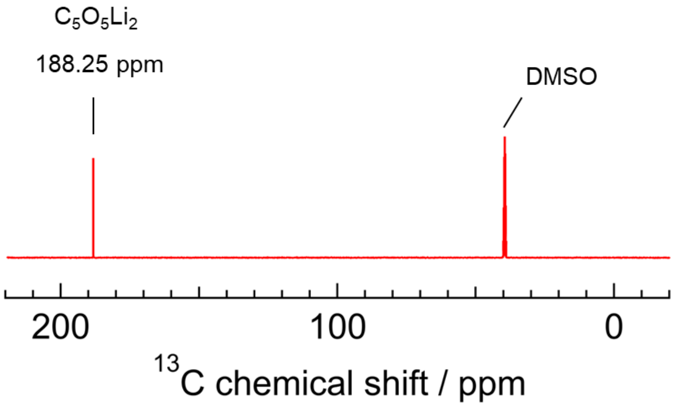

Figure S6. ^{13}C NMR spectrum of $C_5O_5Li_2$ in DMSO-*d6*.

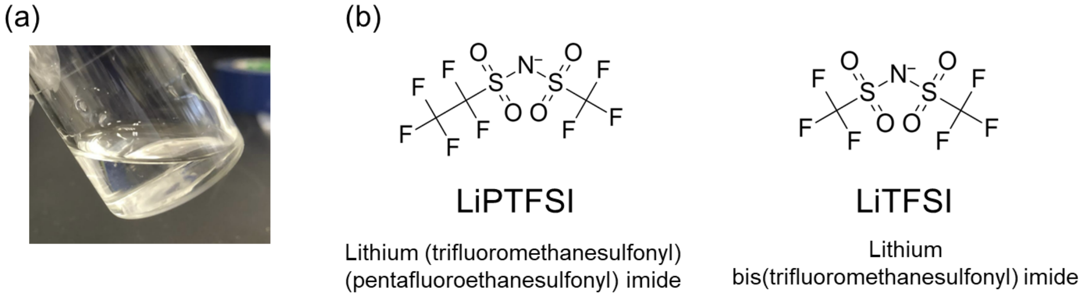

Figure S7. (a) A Photograph of $Li(PTFSI)_{0.6}(TFSI)_{0.4}(H_2O)_{1.2}$, and (b) chemical structures for LiPTFSI and LiTFSI.

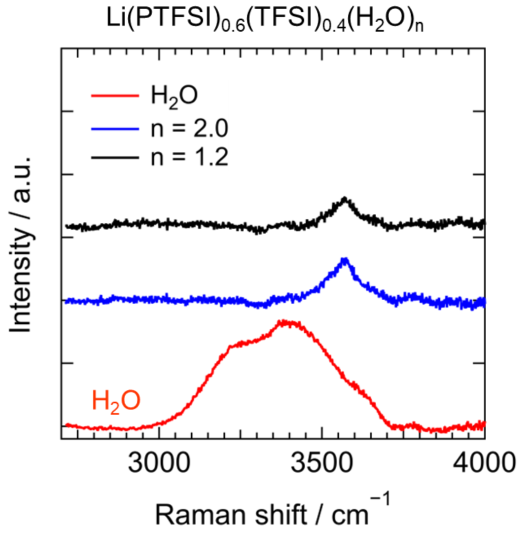

Figure S8. Raman spectra of $Li(PTFSI)_{0.6}(TFSI)_{0.4}(H_2O)_n$ (n = 1.2, and 2.0) and H_2O solvent.

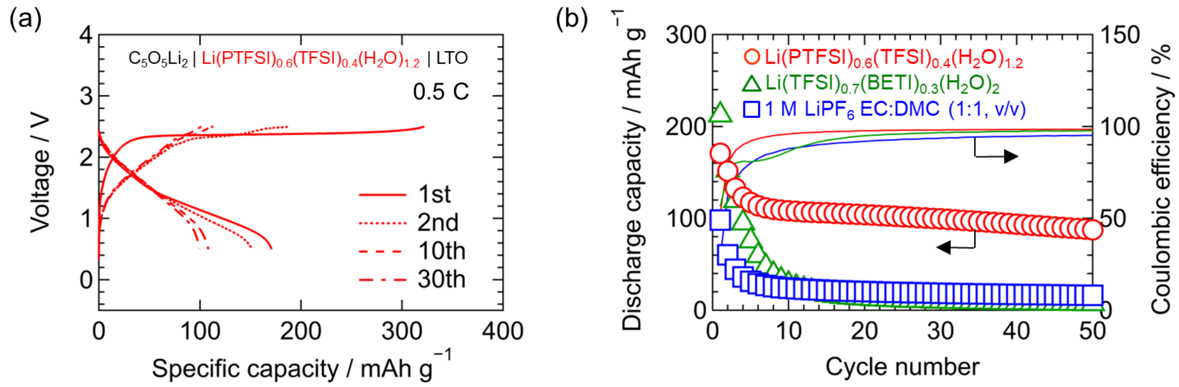

Figure S9. 1st, 2nd, 10th, and 30th charge-discharge profiles of the $C_5O_5Li_2$-based organic LIBs with $Li(PTFSI)_{0.6}(TFSI)_{0.4}(H_2O)_{1.2}$, and (b) cycle performance of the cells with $Li(PTFSI)_{0.6}(TFSI)_{0.4}(H_2O)_{1.2}$, $Li(TFSI)_{0.7}(BETI)_{0.3}(H_2O)_2$, and 1 M $LiPF_6$ EC:DMC at a C-rate of 0.5 C.

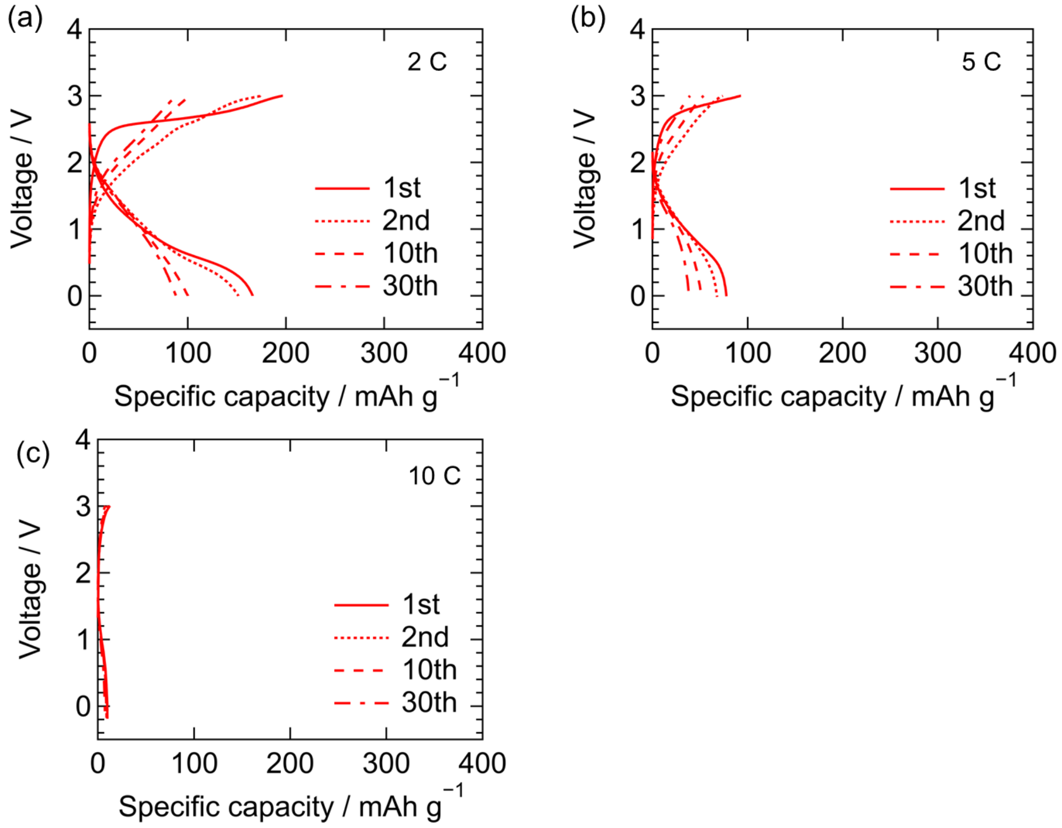

Figure S10. (a) 1st, 2nd, 10th, and 30th charge-discharge profiles of the $C_5O_5Li_2$-based organic LIBs with $Li(PTFSI)_{0.6}(TFSI)_{0.4}(H_2O)_{1.2}$ at C-rates of (a) 2 C, (b) 5 C, and (c) 10 C.

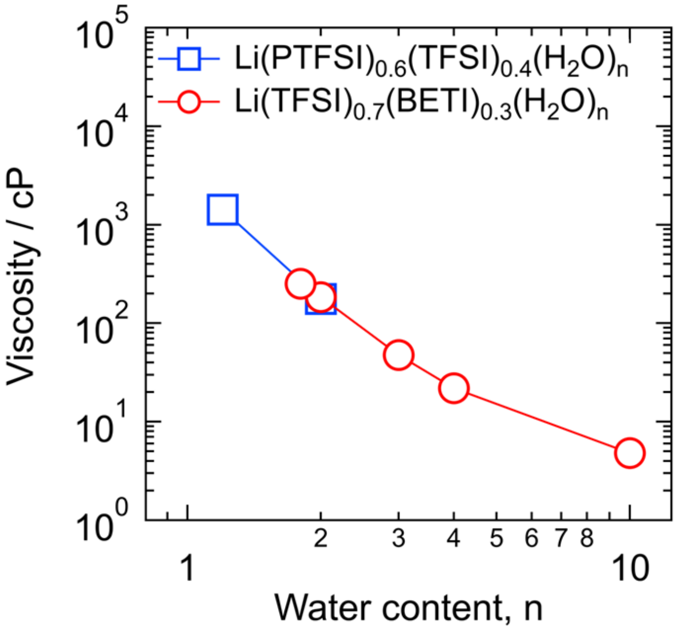

Figure S11. Viscosities of the aqueous electrolytes of $Li(PTFSI)_{0.6}(TFSI)_{0.4}(H_2O)_n$ (n = 1.2, 2.0), and $Li(TFSI)_{0.7}(BETI)_{0.3}(H_2O)_n$ (n = 1.8, 2.0, 3.0, 4.0, 10) at 25°C.

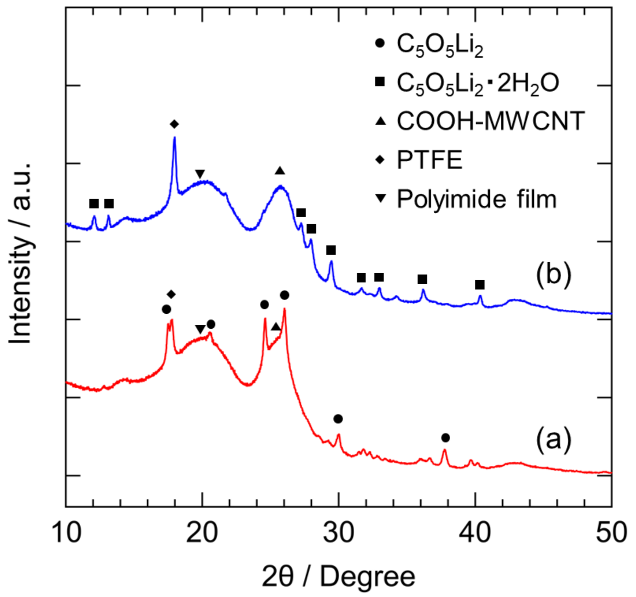

Figure S12. Ex situ XRD patterns of $C_5O_5Li_2$ electrode sheet (a) before and (b) after 2 cycles charge-discharge measurements.

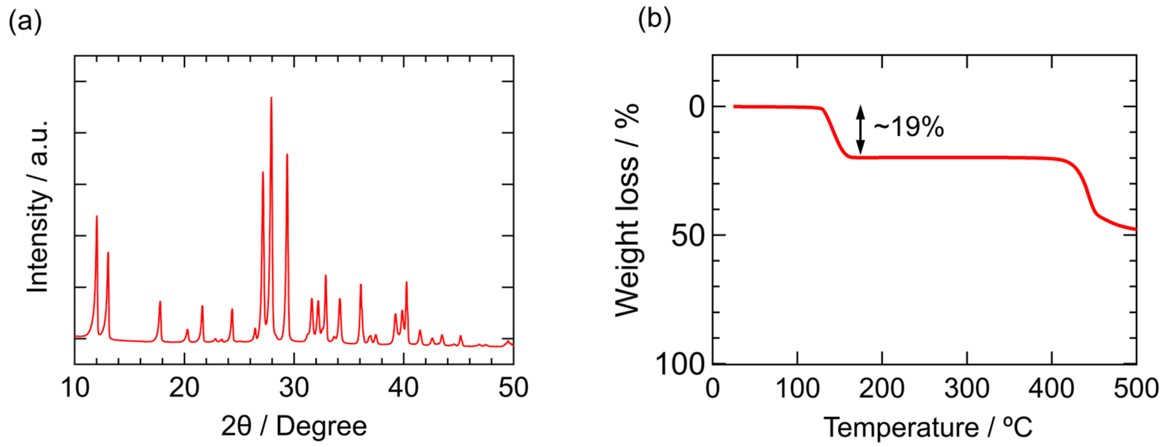

Figure S13. (a) XRD pattern and (b) Thermogravimetric curve of $C_5O_5Li_2$ hydrate powder without vacuum-drying at 150°C.

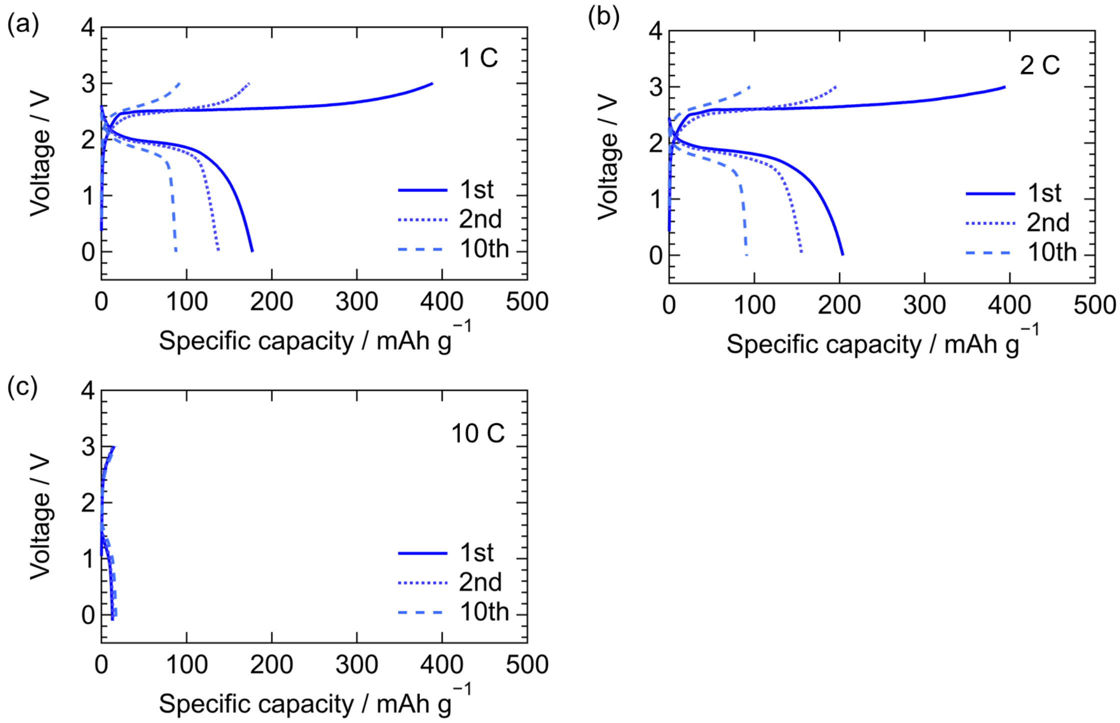

Figure S14. 1st, 2nd, and 10th charge-discharge profiles of the $C_5O_5Li_2$-based organic LIBs with 1 M $LiPF_6$ EC:DMC at C-rates of (a) 1 C, (b) 2 C, and (c) 10 C.

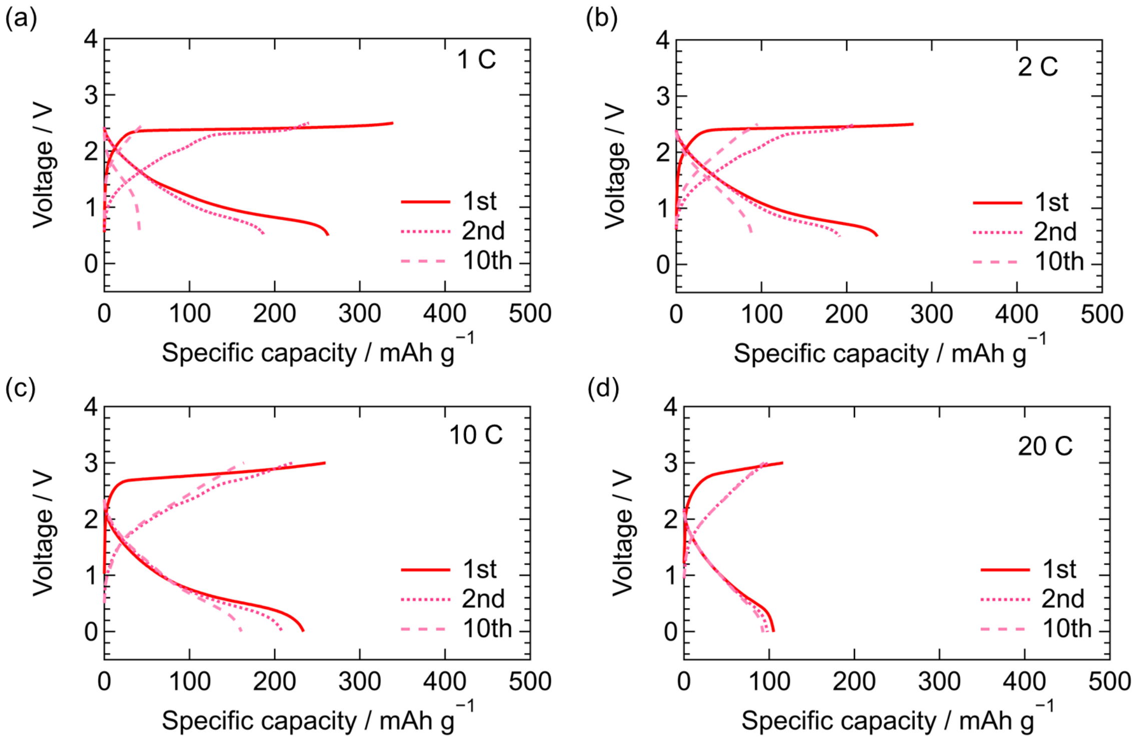

Figure S15. 1st, 2nd, and 10th charge-discharge profiles of the $C_5O_5Li_2$-based organic LIBs with hydrate-melt electrolytes at C-rates of (a) 1 C, (b) 2 C, (c) 10 C, and (d) 20 C.

Table S1. The battery performances of the croconate-based organic batteries in previous researches were summarized. The cell configuration, 1st discharge capacites, and cycle retention ratio were summarized.

Cathode	Electrolytes	Anode	1st discharge capcities / mAh g^{-1}	Cycle retention ratio / %	Battery type	Ref.
$C_5O_5H_2$ dissolved in 1 M $LiPF_6$ GBL	LICGC	Li	102.2 mAh g^{-1}	-	LIBs	1
$C_5O_5Na_2$/COOH-MWCNT/PTFE	saturated $NaPF_6$ PC	Hard Carbon	123 mAh g^{-1} @1 C	41%@1 C, after 30 cycles	SIB	2
$C_5O_5Na_2$/COOH-MWCNT/PTFE	NaFSI/AN = 1:2.7, molar ratio	Hard Carbon	117 mAh g^{-1} @10C	55%@10 C, after 100 cycles	SIB	3
$C_5O_5Li_2$/COOH-MWCNT/PTFE	$Li(TFSI)_{0.7}(BETI)_{0.3}(H_2O)_2$	LTO	105 mAh g^{-1} @20 C	73%@20 C, after 100 cycles	LIBs	This study
$C_5O_5Li_2$/COOH-MWCNT/PTFE	$Li(PTFSI)_{0.6}(TFSI)_{0.4}(H_2O)_{1.2}$	LTO	171 mAh g^{-1} @0.5 C	51%@0.5 C, after 50 cycles	LIBs	This study

References

1. Katsuyama, Y.; Kobayashi, H.; Iwase, K.; Gambe, Y.; Honma, I. Are Redox-Active Organic Small Molecules Applicable for High-Voltage (>4 V) Lithium-Ion Battery Cathodes? *Adv. Sci.* **2022**, *9* (12), 2200187.
2. Gambe, Y.; Kobayashi, H.; Honma, I. A 3.5 V-Class Organic Sodium-Ion Battery Using a Croconate Cathode. *Chem. Eng. J.* **2024**, *479*, 147760.
3. Gambe, Y.; Kobayashi, H.; Honma, I. Acetonitrile-Based Highly Concentrated Electrolytes for High-Power Organic Sodium-Ion Batteries. *ACS Appl. Mater. Interfaces* **2025**, *17* (2), 3316–3323.
